# Characterization of PANoptosis-related genes and the immune landscape in moyamoya disease

**DOI:** 10.1038/s41598-024-61241-w

**Published:** 2024-05-04

**Authors:** Zhenyu Zhou, Yanru Wang, Junze Zhang, Ziqi Liu, Xiaokuan Hao, Xilong Wang, Shihao He, Rong Wang

**Affiliations:** 1 Department of Neurosurgery, Beijing Tiantan Hospital, Capital Medical University, Beijing, 100070 China; 2 Department of Neurosurgery, Peking Union Medical College Hospital, Peking Union Medical College and Chinese Academy of Medical Sciences, Beijing, 100730 China; 3 China National Clinical Research Center for Neurological Diseases, Beijing, 100070 China; 4 Collaborative Innovation Center for Brain Disorders, Beijing Institute of Brain Disorders, Capital Medical University, Beijing, 100069 China

**Keywords:** Bioinformatics, Gene expression analysis, Immunological techniques

## Abstract

Moyamoya disease (MMD) is a cerebrovascular narrowing and occlusive condition characterized by progressive stenosis of the terminal portion of the internal carotid artery and the formation of an abnormal network of dilated, fragile perforators at the base of the brain. However, the role of PANoptosis, an apoptotic mechanism associated with vascular disease, has not been elucidated in MMD. In our study, a total of 40 patients’ genetic data were included, and a total of 815 MMD-related differential genes were screened, including 215 upregulated genes and 600 downregulated genes. Among them, DNAJA3, ESR1, H19, KRT18 and STK3 were five key genes. These five key genes were associated with a variety of immune cells and immune factors. Moreover, GSEA (gene set enrichment analysis) and GSVA (gene set variation analysis) showed that the different expression levels of the five key genes affected multiple signaling pathways associated with MMD. In addition, they were associated with the expression of MMD-related genes. Then, based on the five key genes, a transcription factor regulatory network was constructed. In addition, targeted therapeutic drugs against MMD-related genes were obtained by the Cmap drug prediction method: MST-312, bisacodyl, indirubin, and tropanyl-3,5-dimethylbenzoate. These results suggest that the PANoptosis-related genes may contribute to the pathogenesis of MMD through multiple mechanisms.

## Introduction

Moyamoya disease (MMD) is a cerebrovascular narrowing and occlusive condition characterized by progressive stenosis of the terminal portion of the internal carotid artery and the formation of an abnormal network of dilated, fragile perforators at the base of the brain^1^. Symptoms of MMD include transient ischemic attacks, strokes, neurocognitive impairment and cerebral hemorrhages^2^. Therefore, an urgent need arises for an explicit mechanism of MMD, which can be used to guide improvements in existing treatments of MMD. However, despite extensive investigation, the molecular etiology and pathogenesis of MMD angiopathy remain unclear^3^. Previous research has shown a close relationship between the narrowing of arteries and the proliferation of vascular smooth muscle cells in the progression of MMD^1,4^. In addition, in some vascular diseases, apoptosis related gene abnormalities play a role by affecting smooth muscle cells. Previous studies have reported that lncRNA growth-arrest-specific transcript 5 (GAS5) is able to induce smooth muscle cell apoptosis and the pathogenesis of abdominal aortic aneurysm in Abdominal aortic aneurysm (AAA)^5,6^. Therefore, it is reasonable to hypothesize that a decrease in apoptosis may be associated with the proliferation of vascular smooth muscle cells and contribute to the pathogenesis of MMD.

The programmed cell death (PCD) pathway of apoptosis is a widely studied cell death mechanism executed by caspase-3 and -7, which are downstream of the initiator caspases caspase-8/10 or -9^7^. Among the proposed forms of PCD, pyroptosis, apoptosis, and necroptosis are the most clearly defined. PANoptosis is an inflammatory programmed cell death that can be activated by components that are simultaneously involved in pyroptosis, apoptosis and necroptosis^8^. PANoptosis, which is controlled by a complex termed the PANoptosome, shares key characteristics of apoptosis, pyroptosis, and necroptosis but cannot be accounted for by any of them alone^9^. Recent trends in PANoptosis have led to a proliferation of studies of altered genes in tumors^10–12^. However, within vascular disease, comparably little attention has been given to possible roles for PANoptosis^13,14^. Previous studies have suggested that apoptosis and smooth muscle cell (SMC) proliferation may play an important role in arterial remodeling in MMD^15^. Although evidence for the effects of PANoptosis on MMD is scarce, the three PCD pathways have been implicated in vascular disease, such as apoptosis affecting vascular smooth muscle cell (VSMC) transformation, influencing the proliferation of vascular endothelial cells and promoting vascular remodeling^6,16–19^.

Therefore, in this study, we explored the relationship between PANoptosis and MMD to discover the possible potential pathogenesis of MMD.

To explore possible pathogenic mechanisms, we downloaded moyamoya disease-related datasets (GSE189993 and GSE 141024) from the GEO database and screened for differential genes between the control and disease groups. Subsequently, we extracted pyroptosis, apoptosis and necrotic apoptosis-related genes from the Gene Cards database as PANoptosis-related genes. The intersection genes of apoptosis-related genes and differential genes were obtained. Differential genes and apoptosis-related genes were then extracted for crossover to find differentially expressed PANoptosis-related genes. In addition, machine learning algorithms and differential gene analyses were applied to find key PANoptosis-related genes and their association with immune infiltration and specific signaling pathways were further considered. This provides new insights into a better understanding of the molecular mechanisms of MMD pathogenesis.

## Results

### Inclusion and differential analysis of gene data related to moyamoya disease and PANoptosis

**Figure 1:**
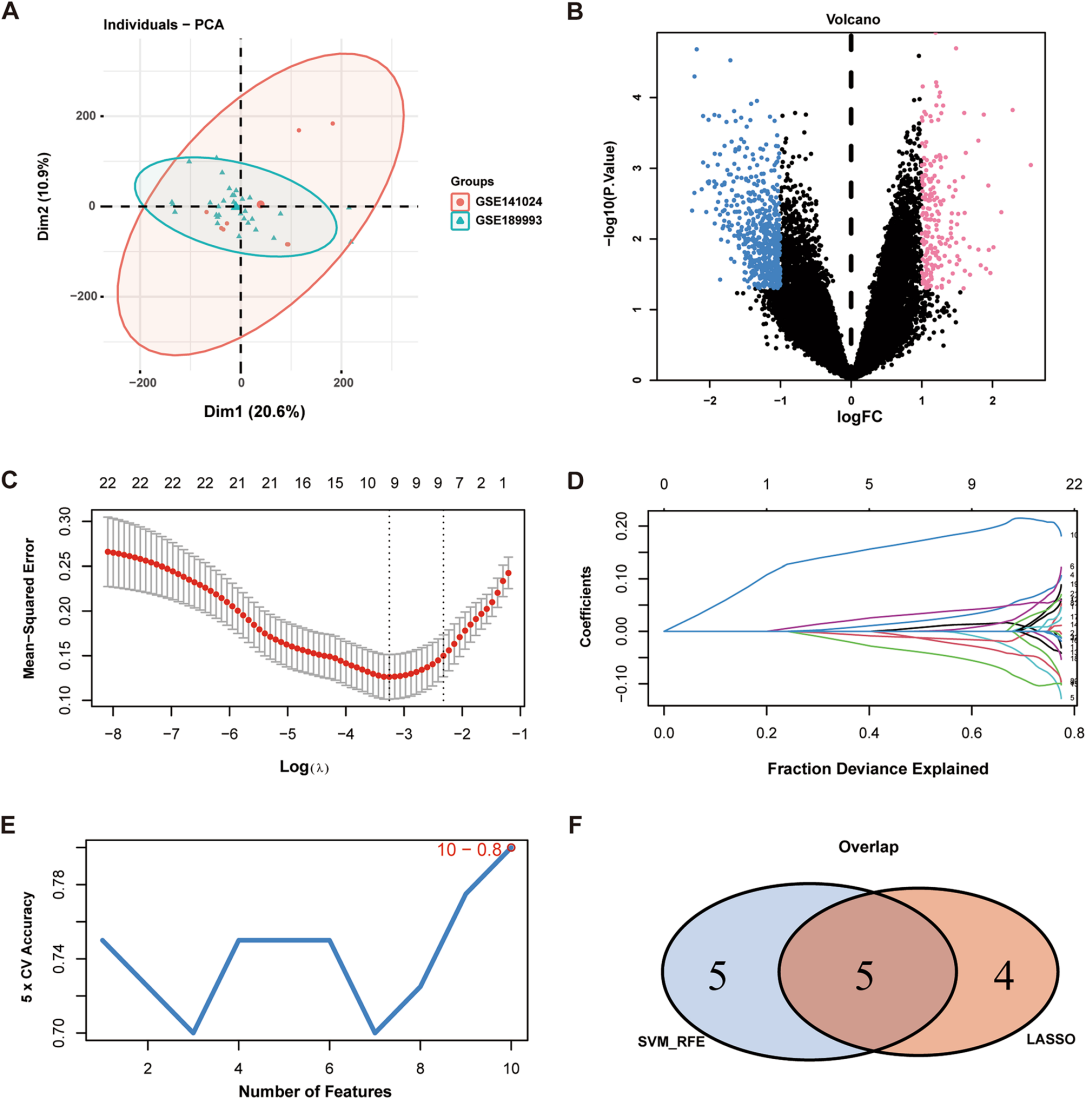
Differential analysis and machine learning of gene datasets. (**A**) Principal component analysis (PCA) plots after correction. (Red: the GSE141024 dataset; Green: the GSE 189,993 dataset). (**B**) Volcano plot of differentially expressed genes between the control group and the disease group. (Red: upregulated genes; Green: downregulated genes. (**C**,**D**) Lasso regression identified 9 genes as characteristic genes for MMD. (**E**), SVM algorithm identifies 10 genes as characteristic genes for MMD. (**F**) Lasso regression and SVM algorithm-screened feature genes take intersection. (Blue: the characteristic genes identified by the SVM algorithm; Red: the characteristic genes identified by Lasso regression). *SVM* support vector machine, *MMD* moyamoya disease.

The datasets GSE189993 and GSE141024 related to moyamoya disease were included from the GEO database, and expression profiling data were included for a total of 40 patients, including the control group (n = 15) and the disease group (n = 25).The microarrays were corrected using the SVA algorithm, and PCA plots were used to demonstrate the batches before and after correction. The results show that the batch effect between chips is reduced after correction by the SVA algorithm (Fig. 1A). The limma package was used to calculate the differential genes between the control group and the disease group (Fig. 1B), and the differential gene screening conditions were P value < 0.05 and |logFC| >  1. A total of 815 differential genes were screened out, of which 215 were upregulated genes and 600 were downregulated genes. Genes with a relevance score > 5 for cellular pyroptosis, apoptosis, and necroptosis were extracted as PANoptosis-related genes through the GeneCards database (https://www.genecards.org/), and 22 intersecting genes were extracted from the intersection of PANoptosis-related genes with differential genes.

### Machine learning to identify PANoptosis-related genes

To further identify the key genes affecting moyamoya disease, lasso regression and SVM algorithms were used to screen the intersecting genes obtained in the previous step to identify the characteristic genes in moyamoya disease, and the results showed that a total of 6 genes were identified by lasso regression as characteristic genes in moyamoya disease (Fig. 1C,D). The results showed that the top 10 characterized genes with the highest screening accuracy in the moyamoya disease dataset (Fig. 1E) intersected with the characterized genes screened by the Lasso regression algorithm, and a total of 5 intersecting genes were screened (Fig. 1F), which were used as key genes in our subsequent study: DNAJA3, ESR1, H19, KRT18 and STK3. Among them DNAJA3, ESR1 and STK3 are down-regulated genes, H19 and KRT18 are up-regulated genes.

### Relationship between key genes and immune infiltration in moyamoya disease

**Figure 2:**
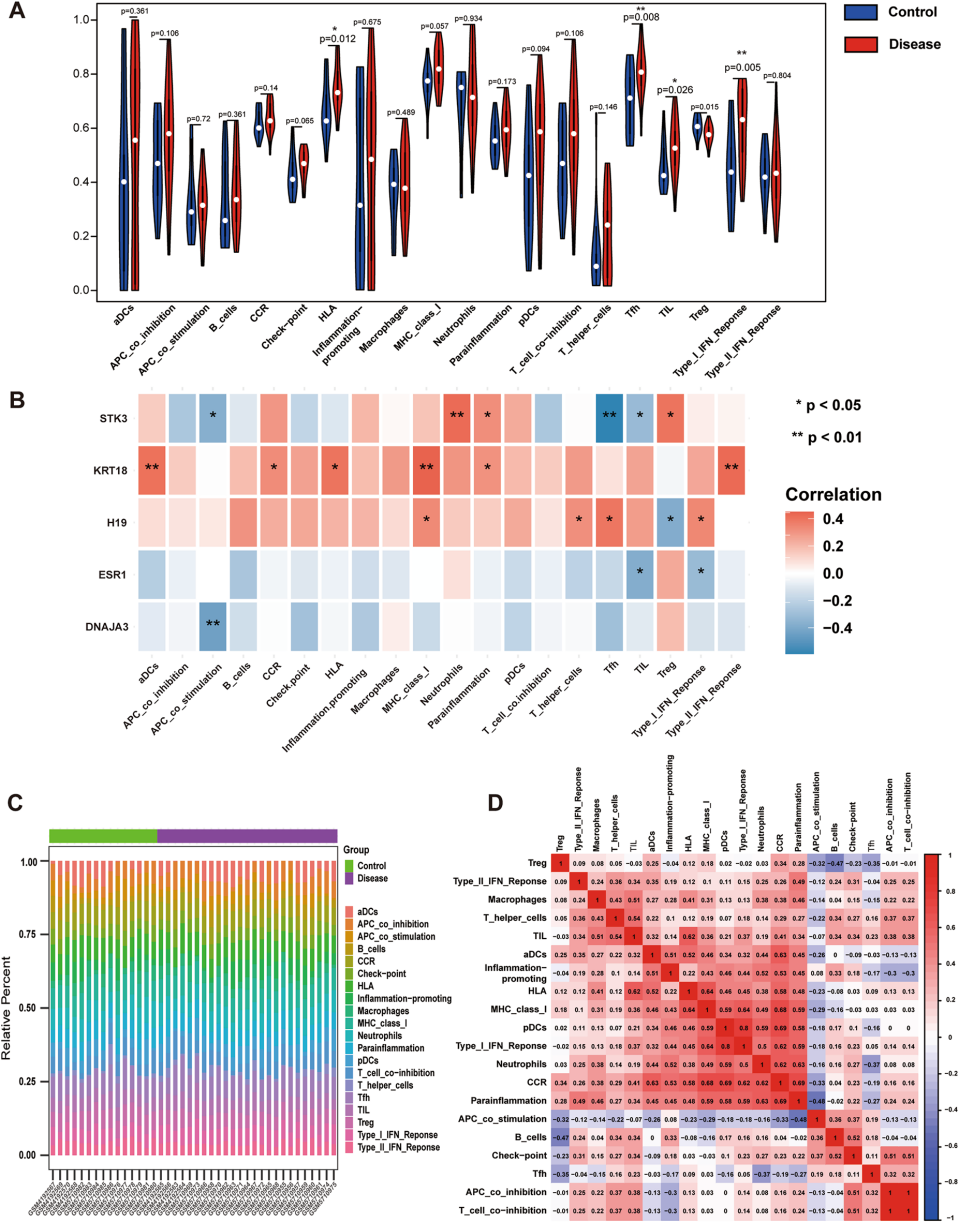
Composition of immune infiltrating cells in association with five key genes. (**A**) Comparisons of immune cells between the control group and disease group. (Blue: the control group; Red: the disease group). (**B**) Map for the correlations between five key genes (DNAJA3, ESR1, H19, KRT18, STK3) and immune infiltrating cells. (*represents P < 0.05, **represents P < 0.01; the redder the color, the stronger the positive correlation; the deeper of the purple color, the stronger the negative correlation). (**C**) Percentage of immune cells between the control group and the disease group. (Green: control group; Purple: disease group). (**D**) Interaction analysis among 20 different immune cells in MMD patients. *MMD* moyamoya disease, *DNAJA3* DnaJ homolog subfamily A member 3, *ESR1* Estrogen receptor alpha, *H19* LncRNA H19, *KRT18* keratin 18, *STK3* Ser/Thr kinase 3.

The microenvironment is mainly composed of a combination of immune cells, extracellular matrix, multiple growth factors, inflammatory factors, and specific physicochemical features, which affects the diagnosis of the disease and sensitivity to clinical treatment. To further explore the potential molecular mechanisms by which key genes influence the progression of moyamoya disease, we analyzed the relationship between key genes and immune infiltration in the moyamoya disease dataset. HLA, Tfh, TIL, and Type I IFN Repons were higher in moyamoya disease patients than in control patients (Fig. 2A). Further exploring the relationship between key genes and immune cells (Fig. 2B), it was found that DNAJA3 was significantly negatively correlated with APC_costimulation; ESR1 was significantly negatively correlated with TIL and Type_I_IFN_Reponse; H19 was significantly positively correlated with MHC_class_I, Tfh, etc., and significantly negatively correlated with Treg; KRT18 was significantly positively correlated with aDCs, MHC_class_I, etc.; and STK3 was significantly positively correlated with neutrophils; KRT18 was significantly positively correlated with aDCs, MHC_class_I, etc.; STK3 was significantly positively correlated with neutrophils, Tregs, etc., and was significantly negatively correlated with Tfh cell, TILs, etc. In addition, the distribution of immune infiltration levels and the correlation of immune cells also showed different forms (Fig. 2C,D). Meanwhile, the correlations between key genes and different immune factors, including immunosuppressive factors, immunostimulatory factors, chemokines and receptors, were obtained from the TISIDB database. These analyses suggested that key genes are closely associated with the level of immune cell infiltration and play an important role in the immune microenvironment (Sup. 1).

### Related signaling pathways of key genes

**Figure 3:**
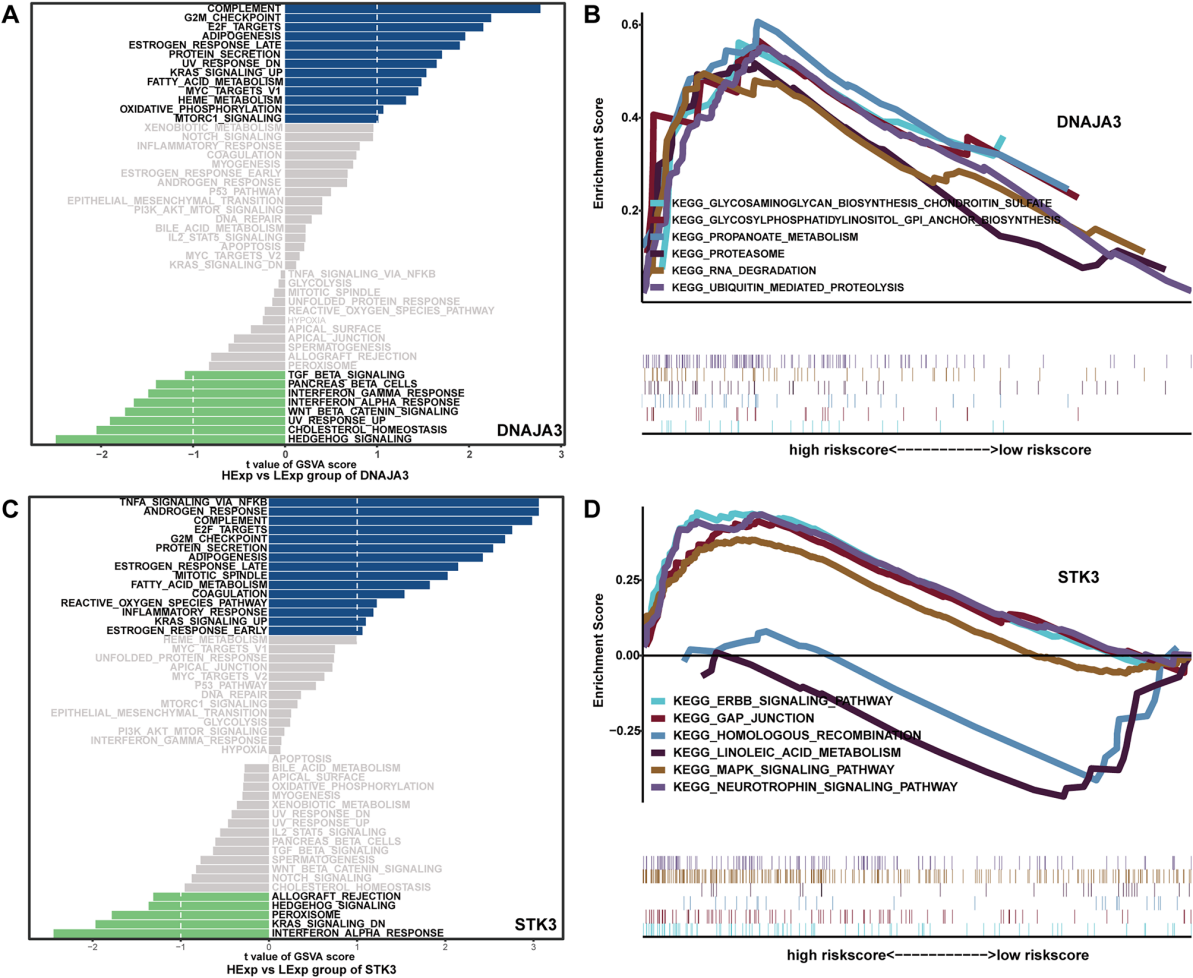
GSVA and GSEA of high and low expression of DNAJA3 and STK3. (**A**) GSVA of DNAJA3. (**B**) GSEA of DNAJA3. (**C**) GSVA of STK3. (**D**) GSEA of STK3. *GSEA* gene set enrichment analysis, *GSVA* gene set variation analysis, *DNAJA3* DnaJ homolog subfamily A member 3, *STK3* Ser/Thr kinase 3.

To study specific signaling pathways enriched for key genes to explore the potential molecular mechanisms by which key genes influence the progression of moyamoya disease. The GSVA and GSEA results showed that high expression of DNAJA3 was mainly enriched in the KRAS signaling pathway, MTORC1 signaling pathway and other signaling pathways. Low expression of DNAJA3 was mainly enriched in the TGFβ signaling pathway, WNT-β CATENIN signaling pathway, HEDGEHOG signaling pathway and others (Fig. 3A). DNAJA3 was enriched in pathways such as PROPANOATE METABOLISM, PROTEASOME, and RNA DEGRADATION (Fig. 3B). High expression of STK3 was mainly enriched in TNFA signaling via the NFKB pathway, KRAS signaling pathway and other signaling pathways. Low expression of STK3 was mainly enriched in the HEDGEHOG signaling pathway, KRAS signaling pathway down and others (Fig. 3C). STK3 was enriched with the ERBB SIGNALING PATHWAY, GAP JUNCTION, MAPK SIGNALING PATHWAY and other pathways (Fig. 3D). High ESR1 expression was mainly enriched in the mitotic spindle, epithelial mesenchymal transition and other signaling pathways. Low ESR1 expression was mainly enriched in the IL2 STAT5 signaling pathway, HEDGEHOG signaling pathway and others (Sup. 2A). ESR1 was enriched in pathways such as DNA REPLICATION, MISMATCH REPAIR, and MTOR SIGNALING PATHWAY. (Sup. 2B); High expression of H19 was mainly enriched in the WNT β-catenin signaling pathway, TGF-β signaling pathway, TNFA signaling via the NFKB pathway and other signaling pathways. Low expression of H19 was mainly enriched in the NOTCH signaling pathway, KRAS signaling pathway down, MTORC1 signaling pathway and others (Sup. 2C). H19 was enriched in pathways such as PROTEASOME, RETINOL METABOLISM, OLFACTORY_ TRANSDUCTION and other pathways (Sup. 2D). High expression was of KRT18 mainly enriched in the WNT β-catenin signaling pathway, TNFA signaling via the NFKB pathway, apoptosis and other signaling pathways. Low expression of KRT18 was mainly enriched in the KRAS signaling pathway, the PI3K AKT MTOR signaling pathway and others (Sup. 2E). KRT18 was enriched with RETINOL METABOLISM, PROPANOATE METABOLISM, STEROID HORMONE BIOSYNTHESIS and other pathways (Sup. 2F).

### Regulatory network analysis of the key genes

**Figure 4:**
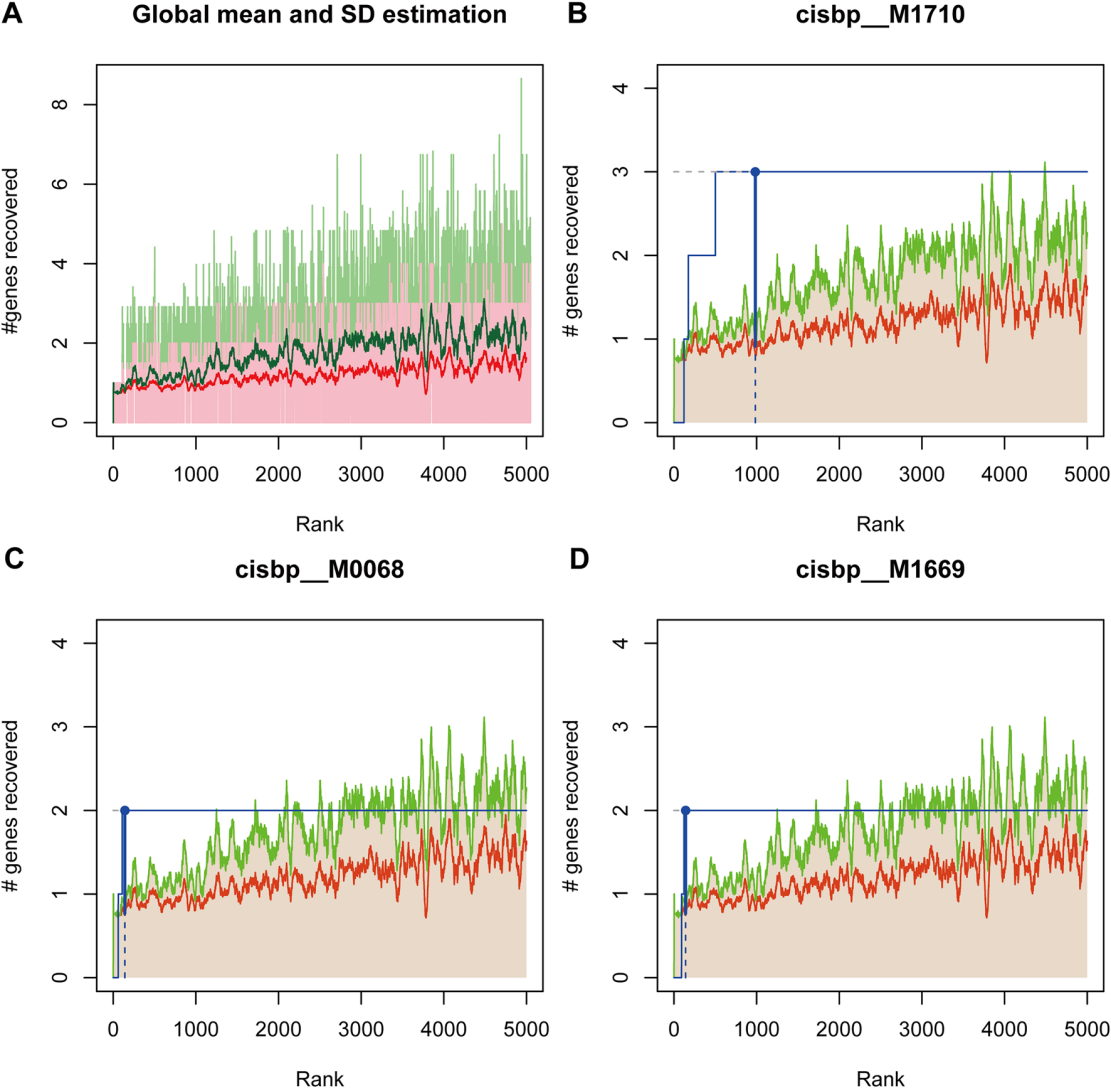
Regulatory network analysis of key genes. (**A**) Enrichment analysis of the transcription factors DNAJA3, ESR1, H19, KRT18, STK3. (**B**) The motif of cisbp_M1710. (**C**) The motif of cisbp_M0068. (**D**) The motif of cisbp_M1669. *DNAJA3* DnaJ homolog subfamily A member 3, *ESR1* estrogen receptor alpha, *H19* LncRNA H19, *KRT18* keratin 18, *STK3* Ser/Thr kinase 3.

Using these five key genes as the set of genes for this analysis, it was found that they are regulated by common mechanisms such as multiple transcription factors. Therefore enrichment analysis was performed for these transcription factors using cumulative recovery curves. Motif-TF annotation, as well as selection analysis of the important genes, showed that the motif with the highest normalized enrichment score (NES: 6.09) was cisbp__M1710. Motifs and corresponding transcription factors for all enriched key genes are shown (Fig. 4). Reverse prediction of the key genes by the miRcode database yielded 82 miRNAs with a total of 208 mRNA-miRNA relationship pairs, which were visualized using Cytoscape (Sup. 3). 3.6. Study of the relationship between five key genes and disease genes associated with moyamoya disease.

**Figure 5:**
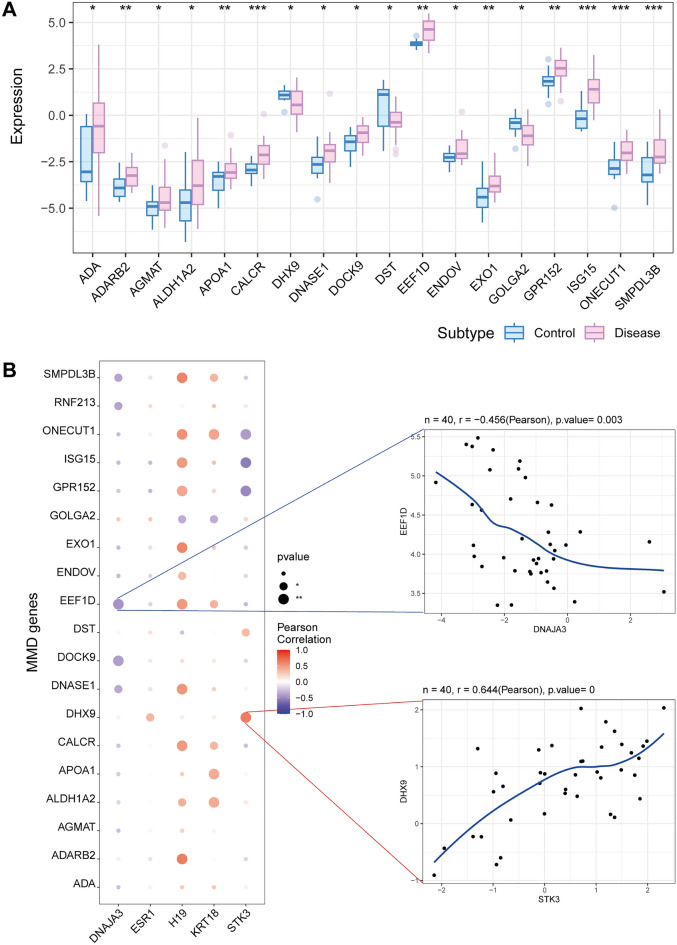
The relationship of key genes and MMD-related genes. (**A**) Genes with significant differences between the control group and the disease group. (*represents P < 0.05, **represents P < 0.01, ***represents P < 0.001). (**B**) Bubble map for the correlations between five key genes and MMD-related genes. (The larger the circle is, the closer the P value is to zero; the redder the color is, the stronger the positive correlation; the deeper the purple color is, the stronger the negative correlation). *MMD* moyamoya disease.

Disease genes associated with moyamoya disease were obtained through the GeneCard database (https://www.genecards.org/). When analyzing the intergroup expression differences of disease genes, the expression of CALCR, ISG15, ONECUT1, and SMPDL3B was found to be significantly different between the two groups of patients (Fig. 5A). Next, the expression levels of five key genes were found to correlate with the expression levels of several moyamoya disease-related genes (Fig. 5B), with H19 positively correlating with ADARB2 (Pearson r = 0.628) and STK3 negatively correlating with ISG15 (Pearson r = − 0.556).

### Screening of drugs targeting PANoptosis in moyamoya disease

Differentially upregulated genes and differentially downregulated genes were categorized into two groups of Top150 and drug prediction was performed using the Connectivity Map database. The results showed that the expression profiles of drug perturbations such as MST-312, bisacodyl, indirubin, and tropanyl-3,5-dimethylbenzoate were negatively correlated with the expression profiles of moyamoya disease perturbations, suggesting that these drugs could attenuate or even reverse the moyamoya disease state.

## Discussion

A comprehensive understanding of the mechanisms of moyamoya disease is key to improving the therapeutic strategy for moyamoya disease. To date, comprehensive research on PANoptosis based on clinical specimens in moyamoya disease is still absent. In addition, moyamoya disease is increasingly recognized as a systemic disease capable of affecting all vasculature of the body rather than a localized stenotic disease affecting only the intracranial vasculature^20,21^. Not only genetic but also immunologic factors play an important role in the systemic vascular alterations in moyamoya disease^22^. Therefore, we systematically investigated the role of PANoptosis -related genes and immune factors in the mechanism of moyamoya disease by bioinformatics analysis.

### Role of key genes in moyamoya disease

DnaJ homolog subfamily A member 3 (DNAJA3), also called Tid1, belongs to the heat shock protein (Hsp) 40 family and serves as a cochaperone and regulatory factor for heat shock protein 70 (Hsp70) tosupport embryonic cell survival, *T*-cell development, muscular development, and apoptosis^23–26^. It was reported that DNAJA3 could affect angiogenesis by regulating the interaction between HIF-1 A and pVHL^27^. It has been demonstrated that low expression of DNAJA3 activates certain signaling pathways related to cell proliferation, migration, and apoptosis, such as the EGFR signaling pathway and the P53 signaling pathway^28^. WNT signaling pathways^29^ and EGFR signaling pathways^30^ are often considered to be closely related to the proliferation and migration of smooth muscle cells. In the present study, downregulation of DNAJA3 was associated with WNT signaling pathways and EGFR signaling pathways. Therefore, it may be one of the causes of abnormal proliferation and migration of vascular smooth muscle cells in moyamoya disease. In addition, as the most potential susceptibility gene of moyamoya disease, the mechanism of RNF213 (Ring Finger Protein 213) in moyamoya disease is still being explored. The negative correlation between DNAJA3 and RNF213 suggests that DNAJA3 may be a gene involved in its mechanism of action in MMD^31^.

Ser/Thr kinase 3 (STK3), also called mammalian sterile 20-like kinase 2 (MST2), is a member of the germinal center kinase group II family, which are mitogen-activated protein kinase (MAPK)-related kinases^32^ As a core component of the Hippo pathway in mammalian cells, MST2 regulates cell proliferation, growth and apoptosis^33^. It has been demonstrated that STK3 inhibition can increase R-155 expression to promote VSMC proliferation^34^. In our study, STK3, a key downregulated gene, was associated with signaling pathways such as the KRAS signaling pathway and HEDGEHOG signaling pathway. Therefore, we can infer that in moyamoya disease, STK3 will promote the proliferation of VSMCs through the KRAS signaling pathway, HEDGEHOG signaling pathway and others in a similar way. In addition, although there was no obvious relationship between STK3 and RNF213, there was a significant negative correlation between STK3 and ISG15(interferon-stimulating gene 15)^35^. ISG15 has been reported to function as a crucial interacting agent of RNF213. This suggests that although stk3 has no direct relationship with RNF213, it can act on RNF213 by affecting ISG15, thereby participating in the pathogenesis of MMD.

In addition, ESR1 is highly expressed in pulmonary artery smooth muscle cells (PASMCs), wherein it increases the proliferation of PASMCs via MAPK and Akt signaling and enhances vascular remodeling^36^. ESR1 tends to decrease vascular smooth muscle cell (VSMC) proliferation through reduced ROS-mediated extracellular signal-regulated kinase (ERK) phosphorylation and smooth muscle cell transition, thereby affecting the progression of MMD^37^.

### Characteristics of immune changes in moyamoya disease

Recent studies have shown that autoimmune inflammation may also be an important pathological mechanism of MMD, but its molecular mechanism is still unclear. Previous studies have used the same gene set and analyzed the characteristics of immune changes in moyamoya disease. UNC13D can reflect the changes of neutrophils in moyamoya disease^38^. In the present study, STK3 also showed a positive correlation with neutrophils. In addition, some studies have compared a variety of immune cells in patients with moyamoya disease and normal controls^39^. Among them, macrophages and mesenchymatous granulocytes were up-regulated in moyamoya disease, which was consistent with the results of this study. These studies on immunity provide new ideas and insights for the future development of immune mechanism and immunotherapy of moyamoya disease.

### Potential small molecule drugs for moyamoya disease

Previous studies have shown that miRNAs and lncRNAs play a key role in the pathogenesis of moyamoya disease^40^. As a bisindole alkaloid, indirubin, which is present not only in Indigo naturalis but also in mollusks, human urine, and various bacteria^41,42^, is one of the main bioactive components of Indigo naturalis. A previous study showed that indirubin showed antiangiogenic activity in an in vivo zebrafish model and an in vitro^28^ HUVEC model^43^. In addition, indirubin derivatives have abundant effects. One study showed the effects of indirubin derivatives on the leukotriene-mediated migration of vascular smooth muscle cells^44^.

### Limitations of the study

However, our study has some limitations and shortcomings. First, the sample size retrieved from the GEO database limits further analysis. The results of the study lack in vivo and in vitro experimental validation.

## Conclusion

In this study, five key genes (DNAJA3, ESR1, H19, KRT18 and STK3) were selected from the MMD and PANoptosis-related genes sets by differential analysis. The functional enrichment analysis of KEGG and GO, the analysis of specific signaling pathways of GSEA and GSVA, and the study of the regulatory mechanisms were investigated to clarify their role in MMD may play a role. The results showed that PANoptosis-related genes related genes may play a role in moyamoya disease by affecting vascular smooth muscle cells, immune cells, immune factors, and mmd susceptibility genes. This study is the first to link PANoptosis with the pathogenesis of MMD, suggesting that PANoptosis-related genes may have potential roles in the pathogenesis of MMD, and providing new ideas and theoretical foundations for the treatment and prevention of MMD.

## Methods

### Datasets and acquisition

All the methods were carried out in accordance with relevant standards and regulations. All the experimental protocols analyzed in this paper using GEO database have been approved by the Ethics Committee of Beijing Tiantan Hospital (KY 2020-045-02). The GEO database (https://www.ncbi.nlm.nih.gov/geo/info/datasets.html), known as GENE EXPRESSION OMNIBUS, is a gene expression database created and maintained by NCBI, the National Center for Biotechnology Information. The Series Matrix File data file of GSE189993, annotated as GPL16699, was downloaded from the NCBI GEO public database, and the expression profile data of 32 patients were included, including 11 cases in the normal group and 21 cases in the disease group. The Series Matrix File data file of GSE141024 was downloaded from the NCBI GEO public database, the annotated file was GPL16699, and the expression profile data of a total of 8 patients were included, including 4 cases in the normal group and 4 cases in the disease group. The information of dataset GSE189993 was obtained from microsamples of the middle cerebral artery (MCA) of MMD patients (n = 21) and control patients (n = 11). The information of dataset GSE141024 was obtained from microsamples of the middle cerebral artery (MCA) and superficial temporal artery (STA) of MMD patients (n = 4) and control patients (n = 4).

### Differential expression analysis by limma

The Limma package is an R package for differential expression analysis of expression profiles, which is used to identify genes that are significantly differentially expressed between groups. The R package "Limma" was used to analyze the differences in the molecular mechanisms of the MMD data, to identify the differentially expressed genes between the control and disease samples, to screen for differentially expressed genes with a P value of 1, and to draw volcano plots and heatmaps of the differentially expressed genes.

### Lasso regression and SVM algorithm for gene selection

Lasso logistic regression and SVM algorithms were used for feature selection of diagnostic markers of disease. The Lasso algorithm uses the “glmnet” software package. In addition, SVM-RFE is a machine learning method based on support vector machines (SVMs), which finds the best variables by removing the feature vectors generated by SVMs and builds a support vector machine model by using the “e1071” software package to further identify the diagnostic value of these biomarkers for the disease.

### Analysis of immune cell infiltration on genes

The ssGSEA method is a widely used method for the evaluation of immune cell types in the microenvironment. The method distinguishes 29 human immune cell phenotypes, including *T* cells, *B* cells, and NK cells. In this study, the ssGSEA algorithm was used to quantify the immune cells in the expression profiles, which were used to infer the relative proportions of the 29 types of infiltrating immune cells, and Spearman correlation analysis was performed on the gene expression as well as the immune cell content.

### GSEA pathway enrichment analysis

GSEA uses a predefined set of genes to rank genes according to their degree of differential expression in two types of samples and then examines whether the predefined set of genes is enriched at the top or bottom of this ranked list. In this study, GSEA was used to compare the signaling pathway differences between the high-expression group and the low-expression group and to explore the molecular mechanism of the key genes in the two groups of patients, in which the number of substitutions was set to 1000 and the type of substitution was set to phenotype.

### Functional enrichment analysis of the PANoptosis-related genes

Functional annotation of intersecting genes was performed using the R package “ClusterProfiler” to comprehensively explore the functional relevance of these intersecting genes. Gene ontology (GO) and Kyoto encyclopedia of genes and genomes (KEGG) were used to assess the relevant functional categories. P values and q-values of less than 0.05 for GO and KEGG-enriched pathways were considered to be significant categories.

### Cmap drug prediction

The connectivity map (CMap) is a gene expression profiling database based on intervening gene expression developed by the Broad Institute; it is mainly used to reveal the functional links between small molecule compounds, genes and disease states. It contains gene microarray data for 1309 small molecule drugs before and after treatment of five human cell lines. The treatment conditions are diverse, including different drugs, different concentrations, and different treatment durations. This study predicts targeted therapeutic drugs for diseases through differentially expressed genes of the disease.

### Statistical analysis

All statistical analyses were performed using the R language (version 4.2.2). All statistical tests were two-sided, and  P < 0.05 was considered statistically significant.

### Supplementary Information

Supplementary Information.

## Data Availability

The datasets for this study can be found in the GEO database (https://www.ncbi.nlm.nih.gov/geo/info/datasets.html) numbered GSE189993, GSE141024.
