# Supplementary material for: Characterization of PANoptosis-related genes and the immune landscape in moyamoya disease
Source: Sci Rep. 2024 May 4;14:10278. doi: 10.1038/s41598-024-61241-w (PMC11069501; doi:10.1038/s41598-024-61241-w)

Supporting information

Table S1: Detailed clinical information for the participants.

ID	disease	Age(year)	sex	Taken medicines	Antibiotics for the operation	Clinical presentation	Duration until surgery from the last clinical presentation	Subtype of MMD	Aneurysm location/size	Epileptic origin
1	MMD	64	F	Cilostazol 100 mg/day, atorvastatin 10 mg/day	Cefazolin 1 g	ICH	2 months	One side	NR	NR
2	MMD	47	F	Aspirin 100 mg/day, Lansoprazole 15 mg/day, Atorvastatin Calcium Hydrate 10 mg/day, Tsumura Goreisan 2.5g/day, Bepotastine Besilate 10 mg/day	Cefazolin 1 g	IF	8 months	Bilateral	NR	NR
3	MMD	48	F	Amlodipine Besilate 5 mg/day	Cefazolin 1 g	ICH	6 months	Bilateral	NR	NR
4	MMD	49	F	Aspirin 100 mg/day	Cefazolin 1 g	TIA	9 months	Bilateral	NR	NR
5	MMD	59	F	Cilostazol 100 mg/day, Esomeprazole Magnesium Hydrate 20 mg/day	Cefazolin 1 g	IF	11 months	Bilateral	NR	NR
6	MMD	50	F	Cilostazol 100 mg/day, Olopatadine Hydrochloride 5 mg/day	Cefazolin 1 g	TIA	1 month	Bilateral	NR	NR
7	MMD	45	F	Aspirin 100 mg/day, Lansoprazole 15 mg/day, Clopidogrel Sulfate 75 mg/day, Pitavastatin Calcium Hydrate 2 mg/day	Cefazolin 1 g	IF	1 year and 1 month	Bilateral	NR	NR

8	MMD	48	F	Aspirin 100 mg/day, Lansoprazole 15 mg/day, Celecoxib 100 mg/day	Cefazolin 1g	TIA	3 months	Bilateral	NR	NR
9	MMD	51	M	Aspirin 100 mg, Etizolam 0.5 mg/day, Olmesartan Medoxomil 10 mg/day, Azelnidipine 8 mg/day	Cefazolin 1 g	TIA	1 month	Bilateral	NR	NR
10	MMD	53	F	Aspirin 100 mg/day, Lansoprazole 15 mg/day	Cefazolin 1 g	TIA	7 months	Bilateral	NR	NR
11	MMD	43	F	Aspirin 100 mg, /day Lansoprazole 15 mg/day, Telmisartan 40 mg/day	Cefazolin 1 g	TIA	1 year and 2 months	Bilateral	NR	NR
12	MMD	34	F	Aspirin 100 mg/day	Cefazolin 1 g	TIA	5 months	Bilateral	NR	NR
13	MMD	29	F	Levetiracetam 1000 mg/day, Phenytoin 300 mg/day, Sodium Ferrous Citrate 100 mg/day	Cefazolin 1 g	ICH	7 months	Bilateral	NR	NR
14	MMD	32	M	Aspirin 100 mg/day, Lansoprazole 15 mg/day	Cefazolin 1 g	TIA	Several days	Bilateral	NR	NR
15	MMD	7	F	NR	Cefazolin 1 g	TIA	Several days	Bilateral	NR	NR
16	MMD	27	M	Aspirin 100 mg/day, Esomeprazole Magnesium Hydrate 20 mg/day	Cefazolin 1 g	TIA	8 months	Bilateral	NR	NR
17	MMD	5	M	Aspirin 30 mg/day	Cefazolin 0.3 g	IF	3 months	Bilateral	NR	NR
18	MMD	39	M	NR	Cefazolin 1 g	TIA	7 months	Bilateral	NR	NR
19	MMD	11	M	Cilostazol 150 mg/day	Cefazolin 0.5 g	TIA	2 months	Bilateral	NR	NR
20	MMD	46	F	Sodium Ferrous Citrate 50	Cefazolin 1 g	TIA	2 months	Bilateral	NR	NR

				mg/day, Olopatadine Hydrochloride 10 mg/day						
21	MMD	35	F	Pregabalin 225 mg/day, Amitriptyline Hydrochloride 35 mg/day, Desloratadine 5 mg/day	Cefazolin 1 g	TIA	8 months	Bilateral	NR	NR
22	MMD	48	F	Aspirin 100 mg/day, Lansoprazole 15 mg/day, Celecoxib 100 mg/day	Cefazolin 1 g	TIA	3 months	Bilateral	NR	NR
23	MMD	51	M	Aspirin 100 mg, Etizolam 0.5 mg/day, Olmesartan Medoxomil 10 mg/day, Azelnidipine 8 mg/day	Cefazolin 1 g	TIA	1 months	Bilateral	NR	NR
24	MMD	53	F	Aspirin 100 mg/day, Lansoprazole 15 mg/day	Cefazolin 1 g	TIA	7 months	Bilateral	NR	NR
25	MMD	43	F	Aspirin 100 mg, /day Lansoprazole 15 mg/day, Telmisartan 40 mg/day	Cefazolin 1 g	TIA	14 months	Bilateral	NR	NR
26	IA	62	F	Loxoprofen Sodium Hydrate 180 mg/day, Rebamipide 300 mg/day, Brotizolam 0.25 mg/day, Diazepam 2 mg/day	Cefazolin 1 g	NR	NR	NR	ICA/27mm	NR
27	IA	78	F	Etizolam 0.5 mg/day, Tandospirone Citrate 20 mg/day, Kallidinogenase 100 units/day, Magnesium Oxide 660 mg/day	Cefazolin 1 g	NR	NR	NR	Covernous/28mm	NR
28	IA	79	F	Aspirin 100 mg/day, Cilostazol 100 mg/day, Acetaminophen 1200 mg/day, Mecobalamin 1500 μg/day, Benidipine Hydrochloride 4 mg/day, Ezetimibe 10 mg/day, Rosuvastatin Calcium 5 mg/day, Candesartan Cilexetil 8 mg/day	Cefazolin 1 g	Oculomotor nerve palsy	1 month	NR	Cavernous/26mm	NR

29	IA	70	F	Valsartan 160 mg/day, Amlodipine Besilate 10 mg/day, Imidapril Hydrochloride 5 mg/day, Doxazosin Mesilate 2 mg/day, methotrexate 10 mg/week	Cefazolin 1 g	Oculomotor nerve palsy	Several days	NR	Cavernous/20mm	NR
30	IA	65	F	Clopidogrel Sulfate 75 mg/day, Atorvastatin Calcium Hydrate 10 mg/day, L-Carbocisteine 1500 mg, Mecobalamin 1500 μg/day, d-Chlorpheniramine Maleate 6 mg/day, Betamethasone 0.5mg/day, d-Chlorpheniramine Maleate 4 mg/day, Olopatadine Hydrochloride 20 mg/day	Cefazolin 1 g	Oculomotor nerve palsy, head ache	Several days	NR	Cavernous/27mm	NR
31	IA	71	F	Aspirin 100 mg/day, Esomeprazole Magnesium Hydrate 20 mg/day, Valsartan 80 mg/day, Amlodipine Besilate 5 mg/day, Suvorexant 15 mg/day	Cefazolin 1 g	NR	NR	NR	ICA/21mm	NR
32	IA	62	F	Loxoprofen Sodium Hydrate 180 mg/day, Rebamipide 300 mg/day, Brotizolam 0.25 mg/day, Diazepam 2 mg/day	Cefazolin 1 g	None	NR	NR	ICA/27mm	NR
33	IA	78	F	Etizolam 0.5 mg/day, Tandospirone Citrate 20 mg/day, Kallidinogenase 100 units/day, Magnesium Oxide 660 mg/day	Cefazolin 1 g	None	NR	NR	Cavernous/28mm	NR

34	IA	79	F	Aspirin 100 mg/day, Cilostazol 100 mg/day, Acetaminophen 1200 mg/day, Mecobalamin 1500 μ g/day, Benidipine Hydrochloride 4 mg/day, Ezetimibe 10 mg/day, Rosuvastatin Calcium 5 mg/day, Candesartan Cilexetil 8 mg/day	Cefazolin 1 g	ONP	1 month	NR	Cavernous/26mm	NR
35	IA	70	F	Valsartan 160 mg/day, Amlodipine Besilate 10 mg/day, Imidapril Hydrochloride 5 mg/day, Doxazosin Mesilate 2 mg/day, methotrexate 10 mg/week	Cefazolin 1 g	ONP	Several days	NR	Cavernous/20mm	NR
36	EPI	56	M	Levetiracetam 2000 mg/day, Lamotrigine 300 mg/day, Perampanel Hydrate 4 mg/day, Pravastatin Sodium 20 mg/day	Cefazolin 1 g	Seizures	Several days	NR	NR	Temporal lobe
37	EPI	14	M	Lacosamide 300 mg/day, Clobazam 15 mg/day, Perampanel Hydrate 6 mg/day	Cefazolin 1 g	Seizures	Several days	NR	NR	Frontal lobe
38	EPI	20	M	Lacosamide 400 mg/day, Lamotrigine 200 mg/day, Carbamazepine 400 mg/day	Cefazolin 1 g	Seizures	Several days	NR	NR	Frontal lobe
39	EPI	43	F	Carbamazepine 300 mg/day, Atorvastatin Calcium Hydrate 5 mg/day, Fexofenadine Hydrochloride 120 mg/day	Cefazolin 1 g	Seizures	Several days	NR	NR	Temporal lobe

40	EPI	2	M	Levetiracetam 325 mg/day, Zonisamid 16 mg/day	Cefotaxime 0.5 g	Seizures	Several days	NR	NR	Frontal lobe

Abbreviations: MMD, moyamoya disease; F, female; M, male; IA, intracranial aneurysm; EPI, epilepsy; ONP, ocular nerve palsy; ICH, intracerebral hemorrhage; IF, infarction; TIA, transient ischemic attack; ICA, internal carotid artery; MCA, middle cerebral artery; NR, not reported.

Figure S1: Correlation between key genes and immune factors

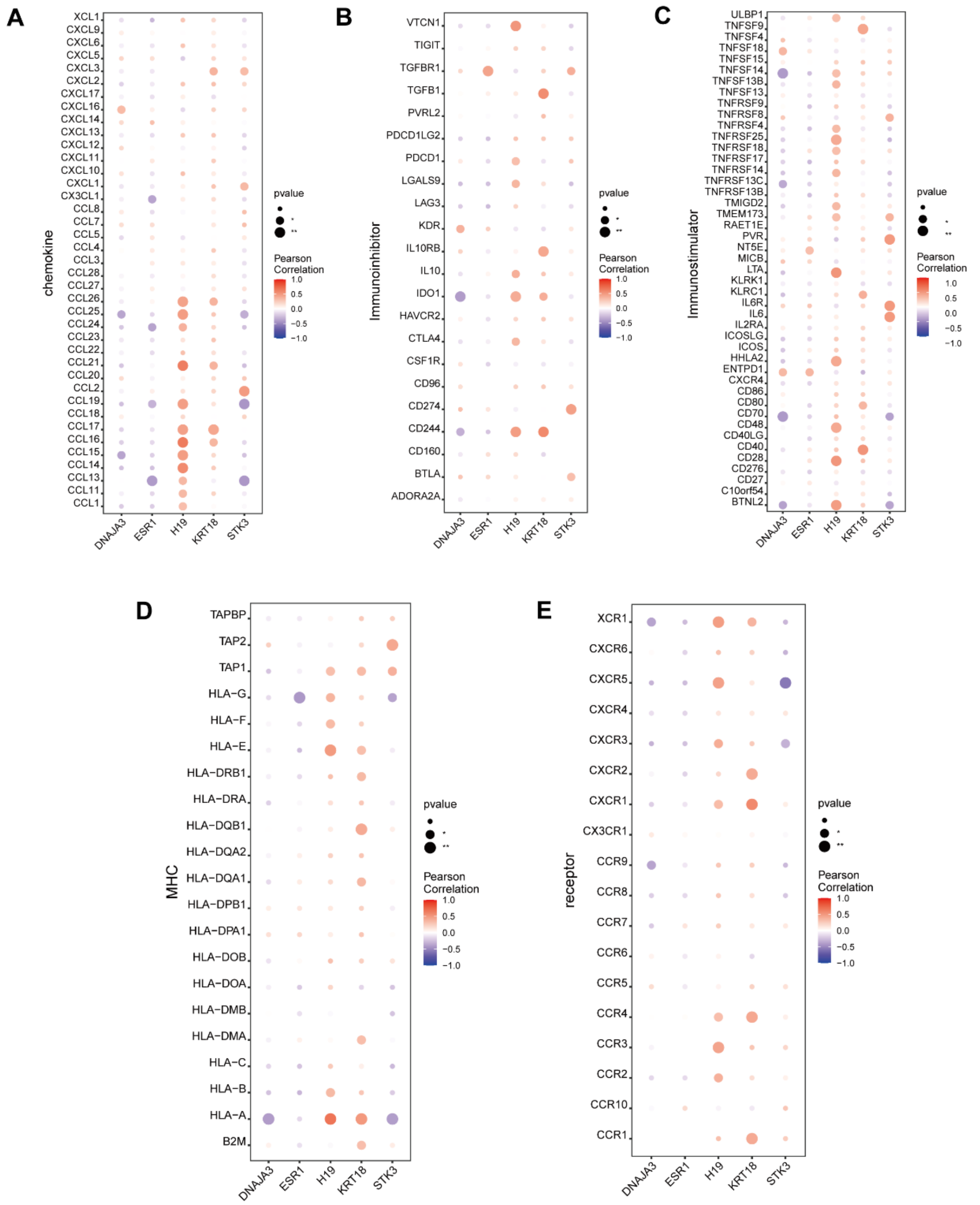

A, Bubble map for the correlations between five key genes and chemokines. (The larger the circle is, the closer the P value is to zero; the redder the color is, the stronger the positive correlation; the deeper of the purple color is, the stronger the negative correlation.)

B, Bubble map for the correlations between five key genes and immunoinhibitors. (The larger the circle is, the closer the P value is to zero; the redder the color is, the stronger the positive correlation; and the deeper the purple color is, the stronger the negative correlation.)

C, Bubble map for the correlations between five key genes and immunostimulators. (The larger the circle is, the closer the P value is to zero; the redder the color is, the stronger the positive correlation; and the deeper of the purple color is, the stronger the negative correlation.)

D, Bubble map for the correlations between five key genes and MHC. (The larger the circle is, the closer the P value is to zero; the redder the color is, the stronger the positive correlation; and the deeper the purple color is, the stronger the negative correlation.)

E, Bubble map for the correlations between five key genes and receptors. (The larger the circle is, the closer the P value is to zero; the redder the color is, the stronger the positive correlation; and the deeper the purple color is, the stronger the negative correlation.)

Figure S2: GSVA and GSEA of high and low expression of ESR1, H19 and KRT18.

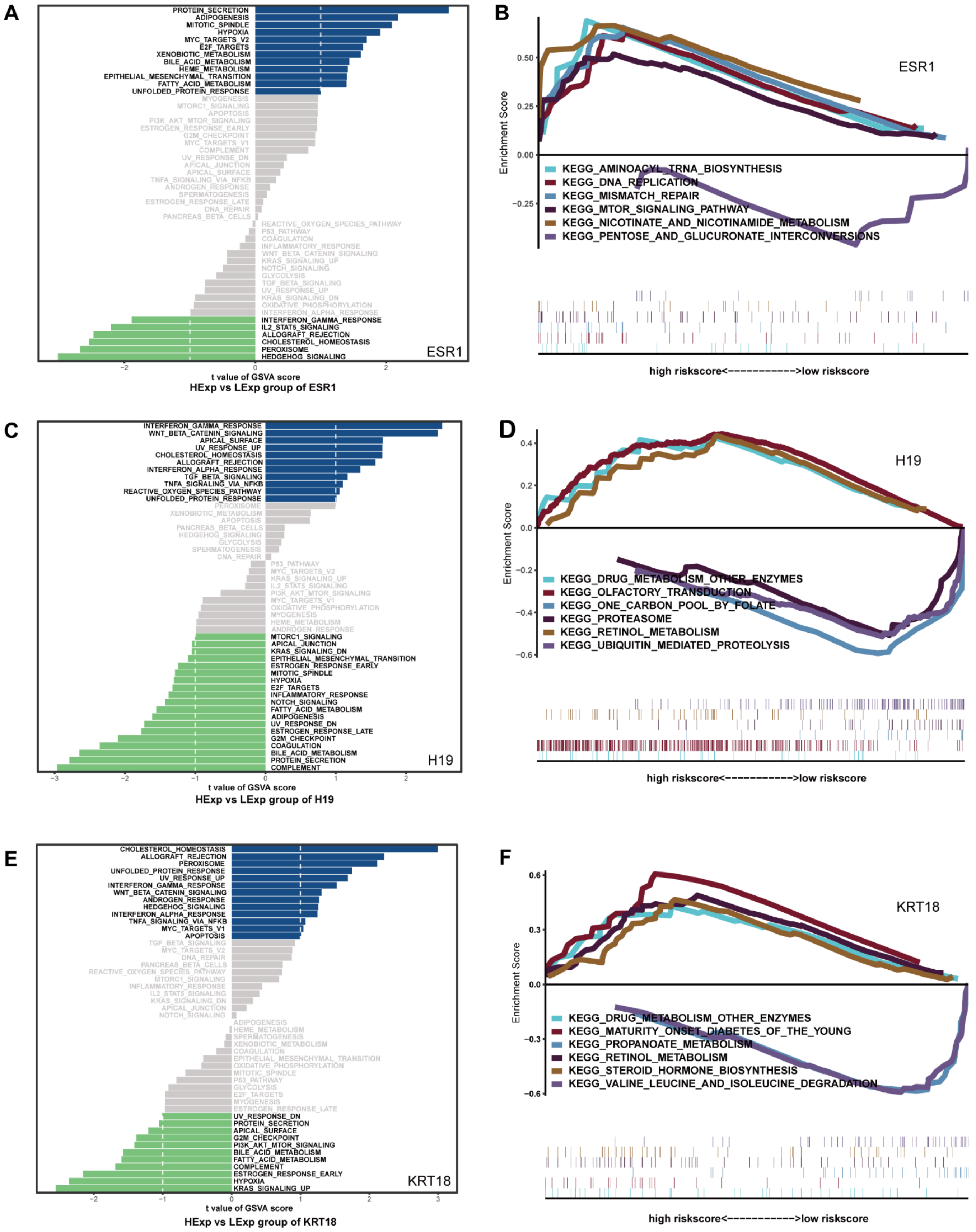

D, GSEA of H19.

E, GEVA of KRT18.

F, GSEA of KRT18.

Figure S3: Meshwork of mRNA-miRNAs for five key genes.

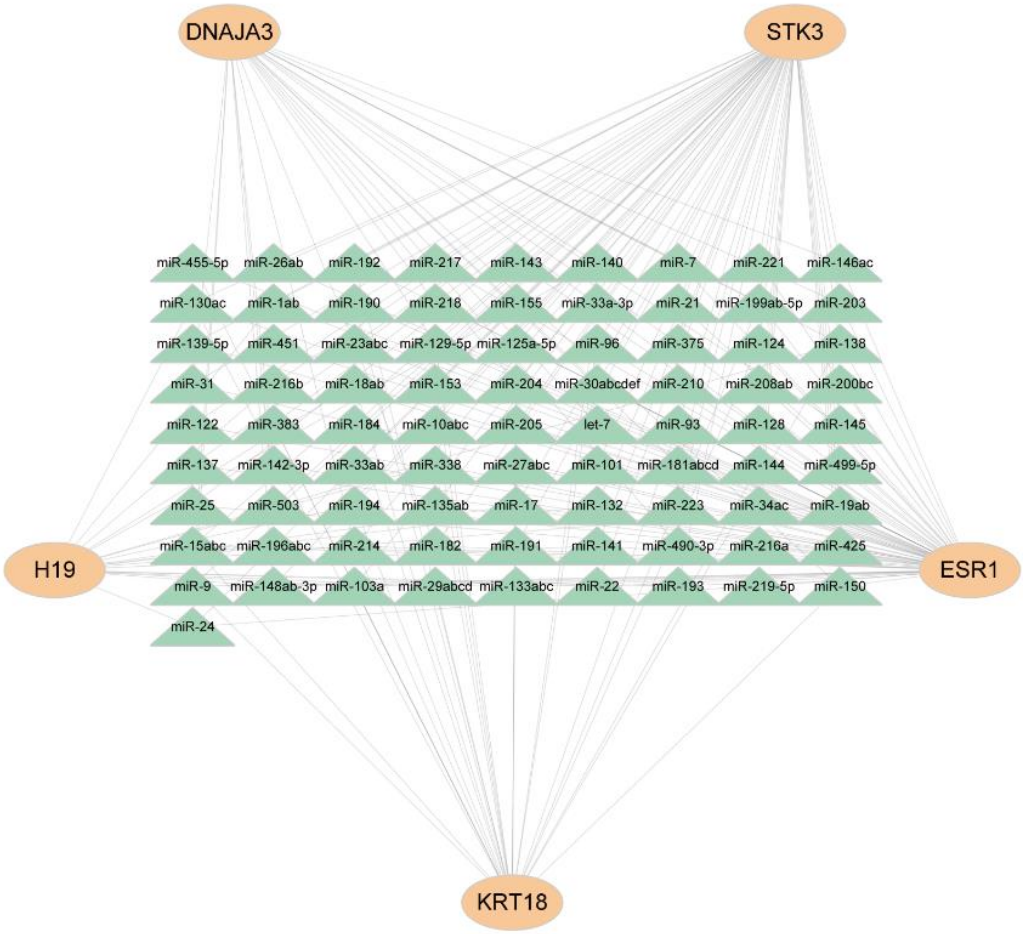

Supplement: Supplementary file 1 — Supplementary Information. [file 41598_2024_61241_MOESM1_ESM.pdf]
